# Identification of Insertion and Deletion (InDel) Markers for Chickpea (*Cicer arietinum* L.) Based on Double-Digest Restriction Site-Associated DNA Sequencing

**DOI:** 10.3390/plants13172530

**Published:** 2024-09-09

**Authors:** Duygu Sari

**Affiliations:** Department of Field Crops, Faculty of Agriculture, Akdeniz University, 07070 Antalya, Turkey; duygusari@akdeniz.edu.tr

**Keywords:** chickpea, insertion, deletion, marker, ddRADSeq

## Abstract

Enhancing the marker repository and the development of breeder-friendly markers in chickpeas is important in relation to chickpea genomics-assisted breeding applications. Insertion–deletion (InDel) markers are widely distributed across genomes and easily observed with specifically designed primers, leading to less time, cost, and labor requirements. In light of this, the present study focused on the identification and development of InDel markers through the use of double-digest restriction site-associated DNA sequencing (ddRADSeq) data from 20 chickpea accessions. Bioinformatic analysis identified 20,700 InDel sites, including 15,031 (72.61%) deletions and 5669 (27.39%) insertions, among the chickpea accessions. The InDel markers ranged from 1 to 25 bp in length, while single-nucleotide-length InDel markers were found to represent the majority of the InDel sites and account for 79% of the total InDel markers. However, we focused on InDel markers wherein the length was greater than a single nucleotide to avoid any read or alignment errors. Among all of the InDel markers, 96.1% were less than 10 bp, 3.6% were between 10 and 20 bp, and 0.3% were more than 20 bp in length. We examined the InDel markers that were 10 bp and longer for the development of InDel markers based on a consideration of the genomic distribution and low-cost genotyping with agarose gels. A total of 29 InDel regions were selected, and primers were successfully designed to evaluate their efficiency. Annotation analysis of the InDel markers revealed them to be found with the highest frequency in the intergenic regions (82.76%), followed by the introns (6.90%), coding sequences (6.90%), and exons (3.45%). Genetic diversity analysis demonstrated that the polymorphic information content of the markers varied from 0.09 to 0.37, with an average of 0.20. Taken together, these results showed the efficiency of InDel marker development for chickpea genetic and genomic studies using the ddRADSeq method. The identified markers might prove valuable for chickpea breeders.

## 1. Introduction

Chickpea (*Cicer arietinum* L.) is one of the most significant legume crops worldwide, although it is mainly produced in India, Australia, Ethiopia, Turkey, and Myanmar [1]. Chickpeas are high in carbohydrates (60–65%), plant-based protein (20–22%), fat (6%), and dietary fiber, especially the soluble fiber raffinose [2]. They also contain several key vitamins and minerals, such as potassium, B-complex vitamins, iron, magnesium, and selenium [3]. Chickpeas are self-pollinated and diploid crops (2n = 2x = 16), and they have a relatively small genome size of ~740 Mbp [3]. Given the ongoing impacts of climate change, it is crucial to enhance agricultural productivity to ensure food security. Thus, the primary objective of plant breeding is to improve high-yield and high-quality varieties. Molecular-marker-assisted breeding helps to achieve these goals in a rapid and efficient manner. Indeed, molecular markers are efficient tools for biodiversity studies, segregation analyses, construction of genetic physical and genetic maps, and transcript profiling [4]. To date, random amplified polymorphic DNA (RAPD) [5,6], amplified fragment length polymorphism (AFLP) [7,8], inter-simple sequence repeat (ISSR) [9,10], and internal transcribed spacer (ITS) [11] markers have been studied in chickpeas. Moreover, in recent years, the development of high-throughput genotyping (or next-generation sequencing [NGS]) has prompted the discovery of high-quality genome-derived simple sequence repeat (SSR) and single-nucleotide polymorphism (SNP) markers in various natural and mapping populations of chickpeas [12,13,14,15]. SSRs are highly informative, abundant in the genome, easy to use, multiallelic, and locus-specific, and they have been widely utilized for plant-breeding applications due to their co-dominance and highly reproducible nature [16,17,18]. However, the unusually high variability and the presence of null alleles do not necessarily reflect patterns of genome-wide genetic diversity [19,20]. In addition, in recent years, SNPs have become important in genetic and genomics applications. SNPs are bi-allelic, co-dominant, and abundantly present in the genome [21,22]. Although several SSR and SNP markers have been developed in chickpeas [10,14,15,23,24,25], the narrow genetic base of cultivated chickpeas might limit the use of these molecular markers in revealing polymorphisms among the genotypes [26], which has inspired efforts to identify alternative markers for chickpea genomics-assisted breeding applications.

Insertion–deletion (InDel) markers are among the main sources of natural variation, and they are widely distributed across the genomes of different plants [27]. InDel markers generally appear due to the movement of transposable elements, unequal crossing over, or replication errors [28,29]. These InDel markers are a preferred alternative to sequence-based markers for genomics-assisted breeding applications. This is due to the desirable genetic features of InDel markers, which SSRs and SNPs also possess [30]. In fact, InDel markers are abundant and widely distributed in the genome, and they are also easily observed with specifically designed primers via simple polymerase chain reaction (PCR) systems and agarose gel electrophoresis, leading to less time, cost, and labor requirements [31,32,33]. So far, InDel markers have been used in some legumes, including common beans [29], lentils [34], and peanuts [35]. However, a small number of studies have investigated the development of InDel markers in chickpeas [27,31,36].

With the aim of enhancing the marker repository and the development of breeder-friendly markers in chickpeas, the present study focused on the identification and development of InDel markers through the use of double-digest restriction site-associated DNA sequencing (ddRADSeq) data from 20 chickpea accessions. The resultant markers were also tested on chickpea germplasm to evaluate their efficiency.

## 2. Results

**Figure 1:**
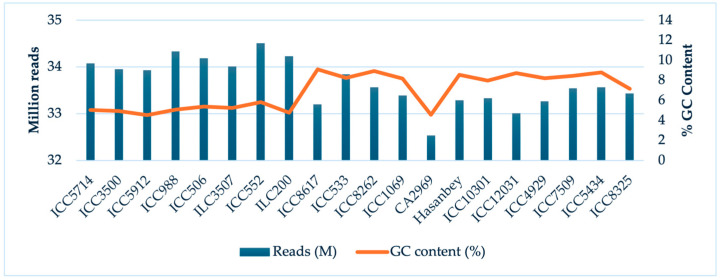
Total number of reads and GC content (%) per accession.

A total of 154.9 M raw sequence reads (with a mean of 7.74 M) were generated using the Illumina HiSeq platform for the 20 chickpea accessions (Table 1). The guanine–cytosine (GC) content of the reads was 33%. The highest number of reads was 11.7 M in ICC552, whereas the lowest was 2.5 M in CA2969 (Figure 1). An overall alignment rate of 97.13% was obtained from the ddRADSeq reads mapped to the chickpea reference genome. Using the variant calling pipeline, 20,700 InDel sites, including 15,031 (72.61%) deletions and 5669 (27.39%) insertions, were identified among the accessions (Table 2). The InDel markers ranged from 1 to 25 bp in length. Single-nucleotide-length InDel markers were found to represent the majority of InDel sites and to account for 79% of the total InDel markers. However, we focused on the InDel markers wherein the length was greater than a single nucleotide to avoid any read or alignment errors. Considering all of the InDel markers, 96.1% were lower than 10 bp, 3.6% were between 10 and 20 bp, and 0.3% were higher than 20 bp in length.

All of the InDel markers were distributed across the eight chromosomes of chickpea, with the highest number being located in chromosome 4 and the lowest number in chromosome 8 (Table 3). The greatest number of insertions and deletions occurred in chromosome 4 and chromosome 6, respectively, whereas the smallest numbers of insertions and deletions were both seen in chromosome 8. The frequency of the InDel markers ranged from 5.49 InDel/Mb (chromosome 8) to 17.92 InDel/Mb (chromosome 4). In light of their genomic distribution and the simple visualization on agarose gels, we examined InDel regions of 10 bp and longer for the identification of InDel markers (Table 4).

There were 689 InDel regions, including 570 (83%) insertions and 119 (17%) deletions, with a length >10 bp examined in the chickpea genome (Table 4). Chromosome 4 exhibited the greatest number of insertions (109) and deletions (25), whereas the smallest number of insertions was observed in chromosome 1 and the smallest number of deletions was noted in chromosome 8. The chromosomal position, size, and sequence information of some insertions and deletions are provided in Tables S1 and S2, respectively. The greatest insertion (25 bp) was identified in chromosome 4 (physical position: 7271444), followed by chromosome 4 (position: 6839631) with 24 bp and then chromosomes 5 (physical position: 44703336), 6 (physical position: 7658552), and 7 (physical position: 33061740) with 23 bp (Table S2). The longest deletion (25 bp) was located in chromosome 2 (position: 7929861) (Table S2).

In total, 29 InDel markers were successfully designed in this study. The InDel markers were dispersed across all eight chromosomes. Figure S1 shows the PCR amplicons generated with the newly designed primers. Annotation analysis of the InDel markers revealed their highest frequency in the intergenic regions (82.76%), followed by the introns (6.90%), coding sequences (CDSs) (6.90%), and exons (3.45%) (Table S3). Moreover, the locus *CA-D-5-385* was found to be related to the *Callose Synthase 10 (CalS10)* gene.

For the genetic diversity analysis of the 20 chickpea varieties, 21 InDel markers were tested in agarose gel, and the bands were scored according to the allele sizes (Table 5). A total of 42 alleles were observed among the accessions. All of the InDel markers exhibited two alleles. The Ne varied between 1.10 and 2.00. Moreover, the highest Ne was obtained in the loci *CA-D-2-357* and *CA-D-4-414.* The average He and uHe values were 0.25 ± 0.04 and 0.26 ± 0.04, respectively. The average I was 0.39 ± 0.04, and the highest I (0.69) was obtained in the loci *CA-D-2-357*, *CA-D-4-414*, and *CA-I-5-413*. In addition, the polymorphism information content (PIC) varied from 0.09 to 0.37, with an average of 0.20. The loci *CA-D-2-357*, *CA-D-4-414*, *CA-I-4-408*, and *CA-I-5-413* presented the highest PIC (0.37), followed by *CA-I-6-531* (0.36) and *CA-D-3-394* (0.35).

**Figure 2:**
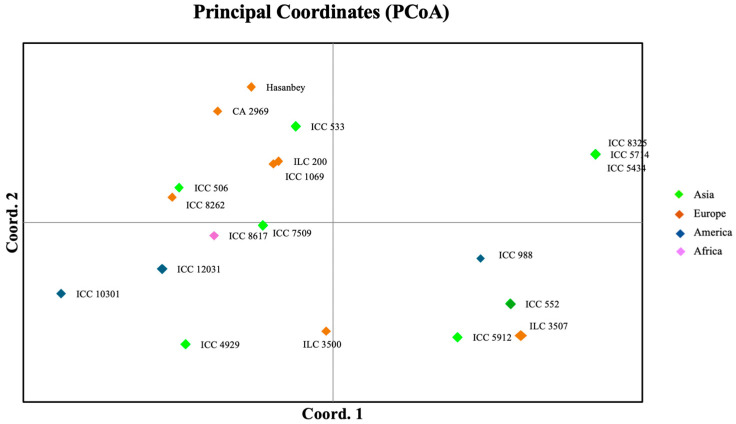
Principal coordinate analysis (PCoA) of the 20 chickpea accessions genotyped with 21 InDel markers.

**Figure 3:**
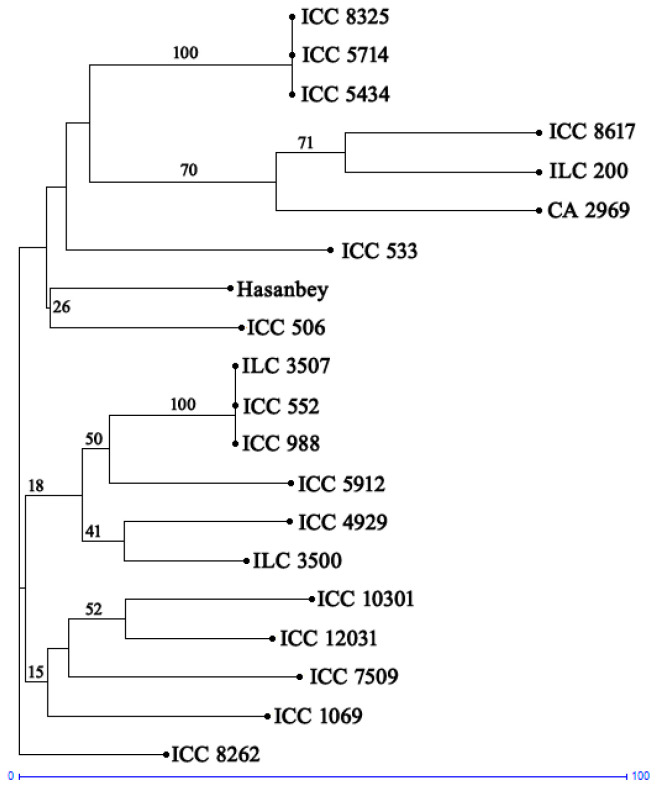
Phylogenetic tree constructed through the neighbor-joining method using InDel markers and 20 chickpea accessions.

According to the PCoA of the molecular data, 25.33% and 18.23% of the total variation was explained by the first and second axis, respectively. Furthermore, the PCoA plots of PC1 versus PC2 using the factorial analysis of GenAlEx showed that the chickpea accessions from the different countries could be divided into three groups without considering their geographic origin (Figure 2). The neighbor-joining tree consisting of 20 chickpea genotypes was constructed with newly developed InDel markers (Figure 3). The tree showed three distinct clusters. Cluster I had nine accessions, mostly originating from Asia. Cluster II was also divided into two sub-clusters and included ten accessions from Asia, America, and Europe. Cluster III had only one accession from Europe.

## 3. Discussion

Climate change is associated with devastating environmental impacts, including extreme weather events (rainfall, droughts), rising sea levels, soil acidification, and the emergence of new pathogenic strains [37,38]. To help overcome these impacts, plant breeders have sought to exploit the natural variations in germplasm resources to develop climate-resilient crops. Molecular markers are important tools for characterizing genetic variation [39]. In the last few decades, the development of PCR-based markers, such as RAPD, AFLP, SSR, and SNP, has served to illuminate the genetic resources of chickpeas [6,8,15]. However, the narrow genetic base of the cultivated chickpea has encouraged breeders to identify alternative markers [40]. In addition, in terms of NGS-accelerated marker identification, ddRADSeq is an NGS protocol based on the development of multiplexed libraries by using two restriction enzymes for the genome complexity reduction [41]. In this study, we identified a total of 20,700 InDel sites using ddRADSeq, which indicated the suitability of this method for identifying InDel regions in the chickpea genome. The identified InDel markers varied among the chromosomes, which further confirmed the effectiveness of this method for genome-wide marker identification in chickpeas. Moreover, the identified markers may be used for biodiversity studies, segregation analyses, and the construction of genetic physical and genetic maps.

Based on the insertions and deletions, different InDel sites were identified among the accessions. The most prevalent type was the single-nucleotide InDel site, similar to the studies conducted in chickpea [31]. An increase in the InDel size caused a decline in the abundance of the InDel markers (Table 2). A negative relationship was observed between the InDel size and abundance, which has previously been reported by Jain et al. [31]. We did not observe any insertions or deletions longer than 25 bp. This could be a flaw on the part of ddRADSeq, as sequencing library preparation using this method can create sequence gaps during assembly [36]. Yang et al. [42] reported that InDel markers longer than 30 bp did not always cause more polymorphism. Our InDel frequency was determined with a density of 35.75 InDel/Mb in the whole genome (20,700 InDel sites in a genome size of 740 Mbp), similar to the frequency observed by Das et al. [27] but lower than the frequency obtained by Jain et al. [31]. The sequencing method, number of genotypes, or bioinformatic parameters applied during the variant calling might have caused these differences in the InDel frequency. When compared with other markers in chickpea, the number of InDel markers was less than the number of SNP markers [24,25,43] and more than the number of SSRs [44], which indicated that the efficiency of the InDel and SNP marker discovery in chickpea was higher than that associated with SSR markers when using NGS. However, due to the cost-effective and simple gel electrophoresis procedures, InDel markers are considered a viable alternative to SNP markers, which are relatively expensive and require a complex platform for genotyping.

All of the identified InDel markers were distributed across the eight chromosomes of chickpea. The greatest number of insertions and deletions was observed in chromosome 4 and chromosome 6, respectively, whereas the smallest numbers of insertions and deletions were both found in chromosome 8. These findings confirmed those of prior studies conducted in chickpeas, which described a large number of markers (SNPs or InDel markers) in chromosome 4 [36,45,46,47,48].

In terms of breeding programs, gel-based markers with breeder-friendly genotyping appear to be a better alternative than SNP or KASP markers when considering the available NGS technologies [49]. This led us to develop 29 agarose-resolvable markers that resulted in successful polymorphic bands among the accessions. Eventually, this provided an effective method for both ddRADSeq library preparation and scripts for InDel identification, resulting in 100% PCR efficiency. In addition, annotation analysis revealed the highest frequency of InDel markers in the intergenic regions (82.76%) (Table S3), with similar results having been observed in different crops [50,51,52]. Moreover, Parida et al. [53] recommended the applicability of polymorphic markers obtained from non-CDS components of chickpeas.

Exploiting genetic diversity is very important when it comes to using and conserving genetic resources and developing new breeding strategies. In the current study, the efficiency of the newly developed InDel markers was evaluated in chickpea germplasm, including twenty accessions from nine different regions in Africa, North America, Asia, and Europe. Analysis of the genetic diversity revealed that the number of Ne varied between 1.10 and 2.00. The average He and uHe values were 0.25 and 0.26, respectively. The average I was 0.39, which is greater than the value obtained by Aggarwal et al. [10]. The high values of genetic diversity parameters, such as Ne, He, uHe, and I, support the validity of these molecular markers for evaluating the genetic diversity of chickpea. The average PIC value of the 21 markers was 0.20, which was greater than the PIC values of the ISSR (0.125) [54] and SNP (0.12) [55] markers, and it was used to develop InDel markers and illuminate the genetic diversity of chickpea. Previous studies also reported higher PIC values for InDel markers in chickpea [36], rice [56], and radish [57]. However, the low PIC value range of the markers was not unexpected, given the limited genetic variation in the chickpea gene pool [58]. Among our results, there were nine InDel markers with PIC values greater than 0.25, accounting for 33.3% of the overall InDel markers. Botstein et al. [59] categorized the PIC values of markers as highly informative (≥0.5), reasonably informative (0.50–0.25), or least informative (≤0.25). Based on this characterization, nine of the markers developed in this study were reasonably informative. The PCoA results and phylogenetic analysis obtained using the 21 InDel markers indicated that the chickpea accessions were uniformly distributed in different groups regardless of their geographic origins. Hence, our findings are similar to those of previous research using RAPD markers [5], where a clear relationship among the accessions was not found.

## 4. Materials and Methods

### 4.1. Plant Material

A total of twenty chickpea accessions from nine different regions in Africa, North America, Asia, and Europe were evaluated for the ddRADSeq analysis in this study (Table 1). The highest number of accessions was from India (8), followed by Spain (3), Turkey (2), the Russian Federation (2), Mexico (2), Iran (1), the United States (1), and Ethiopia (1). The seeds of each accession in the collection were sown in pots for the DNA analysis.

### 4.2. DNA Extraction

The total genomic DNA was extracted from young leaves of the plants using the cetyltrimethylammonium bromide (CTAB) method described by Doyle and Doyle [60] with minor modifications, such as the use of extra chloroform–isoamyl alcohol and 70% ethanol cleaning steps to increase DNA purity. The DNA quality and quantity were checked by means of electrophoresis on 1% agarose gels, and the amount was normalized to 100 ng/μL using lambda DNA as the reference.

### 4.3. Library Preparation, Sequencing, and InDel Calling

The ddRADSeq library was prepared using a modified version of the method reported by Peterson et al. [41], where a reduced representative genomic library was prepared using restriction enzymes (VspI and MspI) via a modification to the ddRAD method. Briefly, each sample (200 ng DNA) was digested with two restriction enzymes—namely, *VspI* and *EcoRI*. Ampure XP beads (Beckman Coulter Genomics, Indianapolis, IN, USA) were used to clean the digestion products, and then P1 and P2 adapter ligations were carried out using T4 ligase buffer. To create an overhang in the VspI restriction cut site, the 3′ end of the P1 adapter was modified. After ligation, 15 cycles of PCR amplification with genotype-specific indexed primers were performed. The PCR products were visualized on agarose gel and then combined and equalized in concentration. The genomic library with an insert size of 400–500 bp was run on the Illumina HiSeq platform (Illumina, San Diego, CA, USA) using the 2 × 150 bp paired-end sequencing protocol. The ddRAD sequencing data of the 20 accessions were deposited in the National Center for Biotechnology Information (NCBI)’s Sequence-Read Archive (SRA) database under accession number PRJNA1064701.

For the bioinformatic analysis, the raw data were demultiplexed with Je software (v1.2) [61]. Quality control and preprocessing of the FASTQ files were performed using fastp [62], and the reads were trimmed by removing those bases with an average Phred score of less than 15. The cleaned data were mapped to the kabuli reference genome 1.0 [3] using the Bowtie 2 aligner (v2.2.6) [63]. Variant calling was performed in Freebayes (Galaxy Version 1.1.0.46-0) [64] with the genotype-specific individual alignment files in BAM format by selecting the following parameters: simple diploid calling with filtering and coverage of 20×. The SNPs were removed from the variant files using VCFfilter (Galaxy Version 1.0.0). The separate.vcf files containing the insertions and deletions were merged into a single data file. The combined variant file was organized in Microsoft Excel 2016 to remove any duplicated regions and arrange the InDel markers based on their sizes. InDel regions of 10 bp and longer were visualized with Integrated Genome Browser (IGB) V9.1.4 [65] using the BAM files of the genotypes and the chickpea reference genome.

### 4.4. Identification of InDel-Flanking Sequences and Primer Design

To develop the genome-wide InDel markers, the flanking sequences of the identified InDel markers were extracted as the target sequence based on the chickpea reference genome using IGB software V9.1.4. For the design of the forward and reverse primers, Primer3Plus (https://www.bioinformatics.nl/cgi-bin/primer3plus/primer3plus.cgi (accessed on 15 March 2024)) [66] was used with the following characteristics: primer length of 18–27 nucleotides, melting temperature of 53–62 °C, GC content of 40–60%, and PCR products of 150–500 bp long. The designed primers were later controlled for possible matches with other loci in the genome. All of the markers were named using the CA-D(I)-X-XXX format, where “CA” stands for chickpea; “D” and “I” stand for deletion and insertion, respectively; “X” stands for the chromosome number; and “XXX” stands for the start of the chromosomal position.

### 4.5. PCR Amplification

A total of 21 InDel markers, with around 3 markers evenly distributed on each chromosome, were selected from the designed primer pairs to be validated on 20 chickpea germplasms. For the PCR analysis, a total volume of 20 of reaction mix was used, which included 1 μL of genomic DNA, 1 μL of 10 × PCR buffer, 2.5 mM MgCl_2_, 0.3 μL of 10 mM dNTP mix, 0.3 μL of each primer (10 μM), 0.2 μL of Taq DNA polymerase (5 U/μL), and double-distilled H_2_O. The PCR reaction was conducted under the following conditions: 95 °C for 2.5 min, followed by 4 cycles of 95 °C for 45 s, 50 °C for 20 s, and 60 °C for 50 s; 30 cycles of 92 °C for 20 s, 50 °C for 20 s, and 60 °C for 50 s; and extension at 60 °C for 10 min [67]. The PCR products were separated on 3% agarose gels, visualized by means of ultraviolet (UV) light, and recorded as codominant data, with genotypes listed by fragment size.

### 4.6. Genetic Diversity Analysis

The calculations of the population genetic parameters, number of alleles (Na), number of effective alleles (Ne), Shannon diversity index (I), expected heterozygosity (He), unexpected heterozygosity (uHe) were performed. Principal coordinate analysis (PCoA) was performed using the GenAlEx V6.5 package [68], and the phylogenetic tree was constructed in DARwin version 5.0 software (https://darwin.cirad.fr/product.php (accessed on 10 May 2024)) using the neighbor-joining (NJ) method and modified in FigTree v1.4.4 (http://tree.bio.ed.ac.uk/software/figtree (accessed on 26 May 2024)).

## 5. Conclusions

The development of NGS technologies has prompted the discovery of high-quality genome-derived markers. InDels are preferred alternative sequence-based markers for genomics-assisted breeding applications. This is due to their desirable genetic features, which SSRs and SNPs also possess. In addition, they can be used in regular laboratories, and they can easily observed with designed primers with less time, cost, and labor via simple PCR systems and agarose gel electrophoresis. In this study, we identified 20,700 InDel sites and developed 29 markers that might play an important role in chickpea genetic and genomic studies. The efficiency of these markers was also tested on 20 different chickpea accessions.

## Figures and Tables

**Table 1:** List of the chickpeas used in the ddRADseq analysis.

No	Species	Kabuli/Desi	Gen Bank Number/Name	Origin	Continent
1	*Cicer arietinum*	Kabuli	ILC 200	Russian Fed	Europe
2	*Cicer arietinum*	Desi	ICC 552	India	Asia
3	*Cicer arietinum*	Kabuli	ILC 3507	Spain	Europe
4	*Cicer arietinum*	Desi	ICC 506	India	Asia
5	*Cicer arietinum*	Desi	ICC 988	Mexico	America
6	*Cicer arietinum*	Desi	ICC 5912	India	Asia
7	*Cicer arietinum*	Kabuli	ILC 3500	Spain	Europe
8	*Cicer arietinum*	Desi	ICC 5714	India	Asia
9	*Cicer arietinum*	Desi	ICC 8325	India	Asia
10	*Cicer arietinum*	Desi	ICC 5434	India	Asia
11	*Cicer arietinum*	Desi	ICC 7509	Iran	Asia
12	*Cicer arietinum*	Desi	ICC 4929	India	Asia
13	*Cicer arietinum*	Desi	ICC 12031	Mexico	America
14	*Cicer arietinum*	Desi	ICC 10301	USA	America
15	*Cicer arietinum*	Kabuli	Hasanbey	Turkey	Europe
16	*Cicer arietinum*	Kabuli	CA 2969	Spain	Europe
17	*Cicer arietinum*	Desi	ICC 1069	Russian Fed	Europe
18	*Cicer arietinum*	Desi	ICC 8262	Turkey	Europe
19	*Cicer arietinum*	Desi	ICC 533	India	Asia
20	*Cicer arietinum*	Desi	ICC 8617	Ethiopia	Africa

**Table 2:** The number and frequency of insertions–deletions identified through double-digest restriction site-associated DNA sequencing (ddRADSeq) analysis of 20 chickpea accessions.

InDel Type	InDel Size (bp)	Number	Frequency (%)
Insertion	1	2843	50.15
	2	655	11.55
	3	377	6.65
	4	294	5.19
	5	221	3.90
	6	192	3.39
	7	164	2.89
	8	134	2.36
	9	124	2.19
	10–20	622	11.00
	>20	43	0.76
	Total	5669	100
Deletion	1	13,589	90.41
	2	681	4.53
	3	240	1.60
	4	135	0.90
	5	57	0.38
	6	76	0.51
	7	51	0.34
	8	35	0.23
	9	30	0.20
	10–20	124	0.82
	>20	13	0.08
	Total	15,031	100

**Table 3:** Distribution of insertions–deletions in the genome.

Chromosome	Number of InDels	Number of Insertions	Number of Deletions	Frequency (InDels/Mb)
Chr1	2715	770	1945	13.11
Chr2	1973	527	1446	9.53
Chr3	2278	544	1734	11.00
Chr4	3710	1250	2460	17.92
Chr5	2695	677	2018	13.02
Chr6	3408	905	2503	16.46
Chr7	2785	704	2081	13.45
Chr8	1136	292	844	5.49

**Table 4:** Distribution of insertions–deletions of length >10 bp in the genome.

Chromosome	Number of Insertions	Number of Deletions
Chr1	33	24
Chr2	60	12
Chr3	67	9
Chr4	109	25
Chr5	84	16
Chr6	90	14
Chr7	91	16
Chr8	36	3
Total	570	119

**Table 5:** Summary of genetic diversity statistics for 20 chickpea accessions.

Marker/Locus	Na *	Ne	I	He	uHe	PIC
*CA-I-1-345*	2.00	1.10	0.20	0.09	0.10	0.09
*CA-D-2-792*	2.00	1.10	0.20	0.09	0.10	0.09
*CA-D-2-357*	2.00	2.00	0.69	0.50	0.51	0.37
*CA-I-2-359*	2.00	1.11	0.21	0.10	0.10	0.09
*CA-I-2-326*	2.00	1.10	0.20	0.09	0.10	0.09
*CA-D-3-394*	2.00	1.83	0.65	0.45	0.47	0.35
*CA-D-3-327*	2.00	1.10	0.20	0.09	0.10	0.09
*CA-I-3-371*	2.00	1.47	0.50	0.32	0.33	0.27
*CA-I-3-142*	2.00	1.10	0.20	0.09	0.10	0.09
*CA-D-4-414*	2.00	2.00	0.69	0.50	0.51	0.37
*CA-D-4-604*	2.00	1.10	0.20	0.09	0.10	0.09
*CA-I-4-408*	2.00	1.95	0.68	0.49	0.50	0.37
*CA-I-4-727*	2.00	1.10	0.20	0.09	0.10	0.09
*CA-D-5-283*	2.00	1.10	0.20	0.09	0.10	0.09
*CA-D-5-385*	2.00	1.34	0.42	0.25	0.26	0.22
*CA-I-5-413*	2.00	1.98	0.69	0.49	0.51	0.37
*CA-I-5-445*	2.00	1.22	0.32	0.18	0.18	0.16
*CA-D-6-397*	2.00	1.36	0.44	0.27	0.27	0.23
*CA-I-6-765*	2.00	1.22	0.32	0.18	0.18	0.16
*CA-I-6-531*	2.00	1.92	0.67	0.48	0.49	0.36
*CA-D-7-330*	2.00	1.34	0.42	0.25	0.26	0.22
Mean	2.00 ± 0.00	1.40 ± 0.08	0.39 ± 0.04	0.25 ± 0.04	0.26 ± 0.04	0.20 ± 0.1

* Number of alleles (Na). Number of effective alleles (Ne). Shannon diversity index (I). Expected heterozygosity (He). Unexpected heterozygosity (uHe). Polymorphic information content (PIC).

## Data Availability

The data presented in this study are available on request from the corresponding author.

## Supplementary Materials

The following supporting information can be downloaded at https://www.mdpi.com/article/10.3390/plants13172530/s1, Table S1. Information about insertions of length >10 bp identified in this study. Table S2. Information about deletions of length >10 bp identified in this study. Table S3. The primer sequences of the 29 InDel markers developed in this study. Figure S1. Amplification of chickpea DNAs with use of selected markers (Ladder 100 bp).
