# Supplementary material for: Identification of Insertion and Deletion (InDel) Markers for Chickpea (Cicer arietinum L.) Based on Double-Digest Restriction Site-Associated DNA Sequencing
Source: Plants (Basel). 2024 Sep 9;13(17):2530. doi: 10.3390/plants13172530 (PMC11397535; doi:10.3390/plants13172530)
Supplement: Supplementary file 1 [file plants-13-02530-s001.zip › Supplementary Files.pdf]

Table S1. Information about insertions of length >10 bp identified in this study.

Chromosome	Physical Position	Size (bp)
Chr1	345689	12
	7653950	18
	1801730	12
	2196431	12
	12673857	15
Chr2	35938514	18
	32644671	16
	30419465	14
	35998161	13
	2816155	12
Chr3	37163693	17
	14207234	16
	38873830	20
	36194243	12
	18402031	16
Chr4	40890559	22
	7271444	25
	6839631	24
	47926782	16
	3428020	17
Chr5	413648	22
	44703336	23
	44583209	13
	9705212	20
	37524904	16
Chr6	7658552	23

	53199805	15
	7856722	14
	28086443	17
	57998181	17
Chr7	33258253	16
	33061740	23
	36714993	17
	14879604	14
	17532939	15
Chr8	8886584	16
	6852982	21
	769796	18
	14704095	12
	6012615	13

Table S2. Information about deletions of length >10 bp identified in this study.

Chromosome	Physical Position	Size (bp)
Chr1	1880585	19
	1995016	19
	32853024	18
	25121975	17
	7884794	15
Chr2	7929861	25
	35768491	22
	27399194	15
	3455389	14
	9775522	17
Chr3	39439344	20

	3276830	14
	8730376	22
	28330211	14
	451560	11
Chr4	4149111	24
	4496263	21
	4301462	18
	41019883	18
	7902059	16
Chr5	28306500	21
	32656992	21
	38571762	18
	37992976	13
	26480855	12
Chr6	31129095	22
	3976058	17
	7985216	17
	1341503	15
	13351266	14
Chr7	33061740	23
	33480779	21
	33214837	12
	23888265	12
	5901243	11
Chr8	11210399	18
	13921474	17
	2260323	14
	5614992	10

Table S3. The primer sequences of the 29 InDel markers developed in this study.

Marker Name	Chromosome	Physical Position	InDel Type	InDel Size (bp)	Forward Primer (5′ to 3′)	Reverse Primer (5′ to 3′)	Product Length (bp)	Locus Location *
CA-D-1-188	chr1	1880585	Deletion	19	TTGTCAAGACTAGAAAACAAGCTGA	AAGCACAAAGTTCTGATTGGA	388	intergenic region
CA-D-1-251	chr1	25121975	Deletion	17	CCCCATTTGCTTGTAGGAGA	CACAGTTGAAAATCACGACCA	464	intergenic region
CA-I-1-345	chr1	345689	Insertion	12	AACTTTGAGGTCTCCGGTGT	TCACTTCCGTTTCTGCCTCT	180	intergenic region
CA-D-2-792	chr2	7929861	Deletion	25	ATTGGATTATGAATGGAATGTATGT	TTGAATATTGTCGCCGGTTC	272	intergenic region
CA-D-2-357	chr2	35768491	Deletion	22	CAATGTGACTGACTGAGGTTTCA	TCAAAAGGAGAAGCGTTTACAA	152	intergenic region
CA-I-2-359	chr2	35938514	Insertion	18	TTTCATTGCTAGGACCACCA	CTTTGTTCCTTTCCGGTCTG	191	CDS
CA-I-2-326	chr2	32644671	Insertion	16	CAATAATAGCCGGAAAGAGTACA	TCGTATCACATTGAAAAATAAAAAGT	167	intergenic region
CA-D-3-394	chr3	39439344	Deletion	20	CAATTAGTACTTCTCTGTTGCCAAA	TTTCAAGAATCGAAGCTCAGG	150	intergenic region
CA-D-3-327	chr3	3276830	Deletion	14	CCGGCAAATGTTTAGTTTATGC	TTGATATTAATTACTACCCCGTCTCA	151	intergenic region
CA-I-3-371	chr3	37163693	Insertion	17	TCAGCTGAGAGGAAGTAATGTTTG	TCTGGCACCTTCTCAGTCAA	151	intergenic region
CA-I-3-142	chr3	14207234	Insertion	16	TCATGCTTTAATTTTTCATTGTTCA	TTTCACTCAAAAATATCCTATAGCTCA	231	intergenic region
CA-D-4-414	chr4	4149111	Deletion	24	CGGTTCCAATTGTGATTGTG	TCAACAATGTCCGAACCAGA	156	intergenic region
CA-D-4-604	chr4	6042518	Deletion	21	AAATTTTCACAATGTTTACTGTGC	AAGTGATTGCAAGACATAAATGC	221	intergenic region
CA-I-4-408	chr4	40890559	Insertion	22	AAAGATGCCTCGTGGTACACT	AATTAAAAGCTGCAATTTGTGC	147	intron
CA-I-4-727	chr4	7271444	Insertion	25	TCCAATGAATGACTTTTGTTTACATT	GGCAATTTCGAACTGTACGC	174	intergenic region
CA-D-5-283	chr5	28306500	Deletion	21	GCTAGCCATCGCCTAAACAA	AAAGGTATAATAACAACTTGACGGGTA	155	intergenic region
CA-D-5-385	chr5	38571762	Deletion	18	CAGATCTTTACCTACAGCCCAAA	CCCATCCGGTAGGGATAAAA	175	CDS
CA-I-5-413	chr5	413648	Insertion	22	AGAAGCCCTGAAAACCACCT	AATGCAATGAGGCATATGTGA	268	intergenic region
CA-I-5-445	chr5	44583209	Insertion	13	TTGGGCAGGCTTCCTATTTT	CAAGTGGTTTCGCTTTGTGA	190	intergenic region
CA-D-6-311	chr6	31129095	Deletion	22	AACGAAGTTTTGAGAAAGGTAATTG	GAAAACCGGATATACCTATATTAGCA	181	intergenic region
CA-D-6-397	chr6	3976058	Deletion	17	CAACATATACTCGCTGTCTCAACA	GAGCTCTACTCCCTTGTTACCG	151	exon
CA-I-6-765	chr6	7658552	Insertion	23	TGAGAGTGAGTGTGGGGTACA	TGTAGTTCGGGTCGTTATAGCC	151	intergenic region
CA-I-6-531	chr6	53199805	Insertion	15	TCACAATACGGAATATAGAAATGTTTG	TCATCAAATTTATAACTCAAATCTTCA	226	intergenic region
CA-D-7-330	chr7	33061740	Deletion	23	TGCAGCTCAAATATTAGAAATGATAGA	TCACACCGTTGGACAGTCAT	159	intergenic region

CA-D-7-338	chr7	33846392	Deletion	12	GCATGATTGAAGCAAAGCAA	GAAAGAGGTTGGAGCACAGC	198	intron
CA-I-7-332	chr7	33258253	Insertion	16	GAAGAATATTCCAAATTCTCCTTAAA	TTTGCTTCTTTGCAAGTAAATCT	180	intergenic region
CA-D-8-112	chr8	11210399	Deletion	18	TGTTTCAGACACTGTTTTAATAATTTG	CAATTGCAATAAAGTCAGAGACATC	216	intergenic region
CA-D-8-139	chr8	13921474	Deletion	17	AAAGCTTAACATGATGACATGAAA	GGTCACTCTTTATAGGACAAGGTG	454	intergenic region
CA-I-8-888	chr8	8886584	Insertion	16	ACCTTAATTTACGATCACTACTTGTCT	CCACAAGGGCTTATGTCTATTG	216	intergenic region

*Coding DNA sequence (CDS)

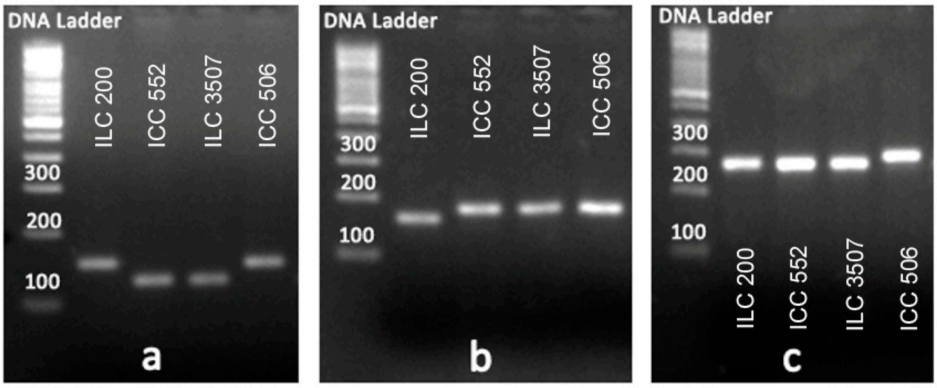

CA-D-4-414 CA-D-5-385 CA-I-5-413

Figure S1. Amplification of chickpea DNAs with use of selected markers (Ladder 100 bp).
